# Structural Changes of Malt Proteins During Boiling

**DOI:** 10.3390/molecules14031081

**Published:** 2009-03-09

**Authors:** Bei Jin, Lin Li, Guo-Qin Liu, Bing Li, Yu-Kui Zhu, Liao-Ning Liao

**Affiliations:** 1 Department of Food Science and Technology, South China University of Technology, Guangzhou 510640, P.R. China; 2 Guangzhou Zhujiang Brewery Group Company, Guangzhou 510315, P.R. China

**Keywords:** Malt variety, Structure, Wort boiling, Protein unfolding, Two-dimensional electrophoresis.

## Abstract

Changes in the physicochemical properties and structure of proteins derived from two malt varieties (Baudin and Guangmai) during wort boiling were investigated by differential scanning calorimetry, SDS-PAGE, two-dimensional electrophoresis, gel filtration chromatography and circular dichroism spectroscopy. The results showed that both protein content and amino acid composition changed only slightly during boiling, and that boiling might cause a gradual unfolding of protein structures, as indicated by the decrease in surface hydrophobicity and free sulfhydryl content and enthalpy value, as well as reduced α-helix contents and markedly increased random coil contents. It was also found that major component of both worts was a boiling-resistant protein with a molecular mass of 40 kDa, and that according to the two-dimensional electrophoresis and SE-HPLC analyses, a small amount of soluble aggregates might be formed via hydrophobic interactions. It was thus concluded that changes of protein structure caused by boiling that might influence beer quality are largely independent of malt variety.

## Introduction

Malted barley is the major source of beer components. The malt quality of a given barley variety is determined by its genetic background and the physical conditions during growth, harvest and storage [1]. A comparative study of malt varieties indicated that in poor ones there were larger amounts of aggregate forms [2], and this aggregated fraction has been found to have a negative effect on beer quality [3]. It was also reported that the rate of decrease of total hordein during malting differed across varieties [4]. Quantitative differences were observed between the protein fractions of Scarlett and Prestige malt varieties, resulting in quantitative differences in the two worts’ protein profiles [5]. All these results seem to confirm that both the content and the distribution of proteins in malt depend on the malt variety and determine beer quality. 

Proteins are essential to the quality of malt and beer. During the malting and brewing processes, a series of changes to the barley proteins occur, including glycation by Maillard reactions during the malting, acylation during the mashing and structural unfolding during the brewing [6]. Moreover, two-dimensional electrophoresis and mass spectrometry have been combined to highlight some barley proteins that could resist the heat treatments during the malting and brewing processes. Most beer proteins in the 10-40 kDa size range [7], which mainly originate from barley proteins, are products of the proteolytic and chemical modifications occurring during the brewing [8]. As expected, from barley to malt and further to beer, most of the heat-stable proteins are disulfide-rich ones [9]. In particular, three major components have been identified in beer, namely a polypeptide with a molecular mass of 40 kDa, known as Protein Z [8,10,11], a 9.7 kDa polypeptide known as LTP1, which is responsible for foam stability [12], and a group of hordein-derived polypeptides (with sizes ranging from 10 kDa to 30 kDa) that are involved in haze formation [13]. Both LTP1 and Z4 are tolerant of high temperatures and resistant to proteolysis, which contribute to their resilience and survival through the brewing process [10,14]. 

As reported by Levis and Young, wort was boiled in a wort boiling “kettle” to inactivate enzymes, remove undesirable flavor components, sterilize the wort, isomerize hop α-acids, and to precipitate haze-forming proteins and polyphenols [15]. It was observed that an increase of the back-pressure on the wort in an external boiler system, might increase the mean boiling temperatures up to 103~110 °C, which accelerated protein coagulation, wort dimethyl sulfide stripping, and the isomerization of hop α-acids. Consequently, the boiling time reduced by 30%-40% [16]. The wort boiling temperature during the brewing process is important for the LTP1 content and formation of the final product (beer); a higher wort boiling temperature (about 102 °C), resulting from the low altitude at sea level, reduces the LTP1 level of beer to 2~3 μg/mL, whereas lower wort boiling temperatures (about 96 °C), resulting from higher altitudes, leads to a LTP1 level of 17~35 μg/mL. In native barley seed, LTP1 gives poor foaming properties. However, it exhibits a foam-promoting form after unfolding during wort boiling. It was found that unfolding would occur in the wort boiling process [6] and glycation might prevent from precipitation due to unfolding during the boiling process. Both glycation and denaturation increase the amphiphilicity of LTP1 polypeptides and contribute to a better adsorption at air-water interfaces of beer foam. [12,14]. Bech *et al.* [17] found that barley LTP1 with a molecular mass of 9.633 kDa was converted to a foam-active form with a molecular mass of 9.6 to 9.99 kDa during wort boiling. Vaag *et al*. [18] also reported a polypeptide of 17 kDa which was rendered foam-active during the mashing and the boiling in a similar manner as reported with LTP1 [12]. All these results confirmed that the boiling process influence beer final composition.

Guangmai, a standard malt variety, is widely used in China for beer production. Baudin, another variety of malt originally from Australia, is now being increasingly employed in China for its good brewing quality. The physico-chemical characterization of final malts reveal that Baudin samples presented lower quantities of total protein and free α-amino nitrogen than Guangmai samples (11.84% and 185 for Guangmai and 11.08% and 132 for Baudin, respectively). It should be pointed out that the total protein quantity of malt is critical for the brewing process [36]. Baudin and Guangmai are new varieties of great commercial interest in the beer production market. Although these two kinds of malts are both used to produce beer in south China, great differences in beer quality exist. In order to seek the reasons for these differences in beer quality, the whole brewing process is being investigated. As the only difference among beers is the difference in the composition of the wort during the boiling, this boiling must be important [7]. Furthermore, information about the changes in physicochemical properties and structure of wort proteins during the boiling process is still scarce. Therefore, this study sought to investigate the effects of boiling process on some physicochemical properties and structure of wort proteins, and reveal the differences in composition and structure of Baudin and Guangmai worts during the boiling process.

## Results and Discussion

### Protein and amino acid analysis

As the changes of the quantity of protein and certain amino acids during the wort boiling process were related to the quality and stability of the final product, this paper sought to examine the changes in the content and composition of proteins and amino acids during the wort boiling process that have not been previously investigated in depth. According to the protein contents of the wort before and after boiling (Table 1), a steady decline in the protein content during the wort boiling process was observed, which matches the results reported by Gorinstein *et al*. [19], but differs from those of Osman *et al*. [20] who reported similar protein contents before and after boiling. 

**Table 1:** Changes of protein contents of the worts during the boiling step^a^.

	Baudin wort		Guangmai wort	
	before boiling	after boiling	before boiling	after boiling
**Protein content (g/L)**	1.261	1.010	1.257	1.080

^a^ Each value is the mean of duplicate measurements.

Moreover, the decline in protein content during the wort boiling was more pronounced for Baudin than for Guangmai wort, revealing a greater effect of boiling on the proteins of the former. As shown in Table 2, after boiling the amino acids content of Baudin wort decreased, while that of Guangmai wort changed only slightly, and that glutamic acid and proline were the two major components of both Baudin and Guangmai, suggesting that hordein is possibly the major component in the wort proteins with the two varieties. Moreover, the two varieties had similar levels of all essential amino acids except leucine and histamine. Furthermore, little difference in wort protein and amino acid contents between the two varieties was observed.

**Table 2:** Changes in amino acid contents of the two wort varieties during boiling^a^.

Amino Acids contents (mg/100g)	Baudin		Guangmai	
	before boiling	after boiling	before boiling	after boiling
Asp	2,905.22	2,265.34	3,016.70	2,904.14
Glu	11,031.11	9,220.12	15,814.40	12,341.07
Ser	2,173.03	1,772.21	3,165.78	2,441.63
Gly	2,311.71	1,856.19	3,077.18	2,456.20
His	1,254.79	1,005.42	1,800.81	1,106.94
Arg	2,124.30	1,878.54	3,426.25	2,278.13
Thr	1,911.44	1,463.98	2,775.93	1,827.74
Ala	1,627.47	1,474.37	2,091.46	1,991.73
Pro	4,247.83	3,509.62	7,777.08	4,969.18
Tyr	1,627.96	1,340.96	2,999.85	1,606.79
Val	2,033.13	1,723.44	3,348.11	2,134.61
Met	816.20	536.48	994.47	518.34
Cys	228.72	150.04	373.23	126.16
Ile	1,359.58	1,073.72	2,229.27	1,401.63
Leu	2,414.81	2,094.16	4,157.57	2,346.27
Phe	1,473.31	1,323.31	2,894.09	1,624.15
Lys	1,043.84	962.62	1,665.08	822.67
Total content	40,584.46	33,650.52	61,607.25	42,897.37

^a ^Each value was the mean of duplicate measurements.

### Emission fluorescence spectroscopy analysis and surface hydrophobicity (H_o_) and SH contents

**Figure 1:**
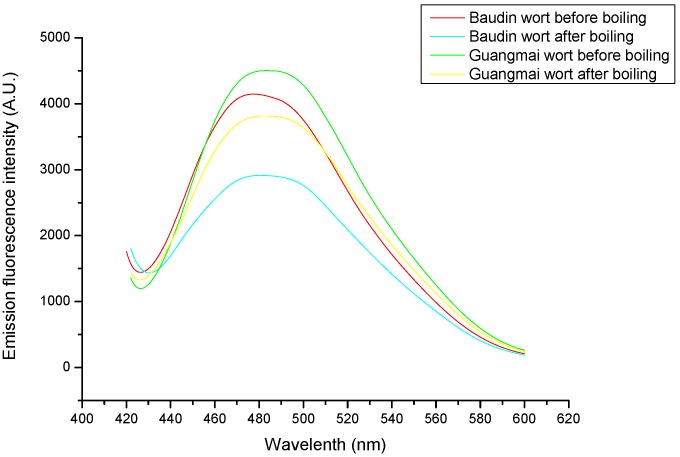
Emission fluorescence intensity spectra of wort proteins before and after boiling with the two varieties (using ANS as a fluorescence probe).

**Figure 2:**
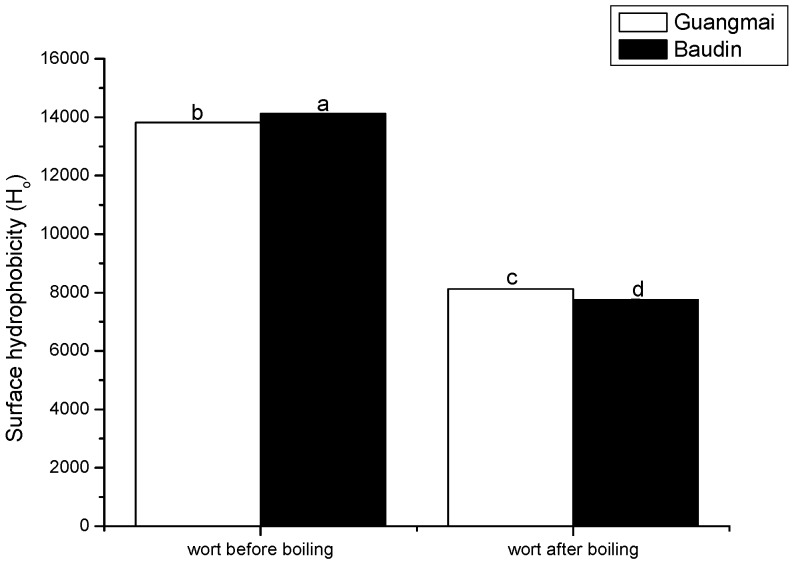
Surface hydrophobicity (H_0_) of wort proteins (before and after boiling) with the two varieties in 10 mmol/L phosphate buffer (pH 7.0). Different characters (a-d) on the top of columns represent significant difference at p<0.05 level during the boiling.

Protein fluorescence originates from the tryptophan/tyrosine residues present in the beer protein [23]. The boiling process caused an obvious decrease in fluorescence intensity (Figure 1), indicating thermal-induced unfolding and association/aggregation of exposed hydrophobic groups. As shown in Figure 2, there were also significant decreases in surface hydrophobicity (H_o_) values during the wort boiling. These results are consistent with the data of fluorescence intensity of the two worts (Figure 1).

Surface hydrophobicity (ANS binding) of proteins may decrease when the proteins unfold as a result of the heating and are then aggregated through hydrophobic interactions, thus reducing the number of ANS binding sites. The surface hydrophobicity was expected to decrease because it is enthalpically favourable [24]. On the other hand, The ANS-binding sites of the wort proteins were possibly rearranged through unfolding during the boiling and ANS did not bind there and thereby led to a decrease in surface hydrophobicity. Moreover, the boiling-induced changes in surface hydrophobicity values (H_o_) were slightly pronounced in the Baudin variety. As a result, we conclude that boiling led to partial unfolding of wort protein and the formation of small amounts of soluble aggregates via hydrophobic interactions.

**Figure 3:**
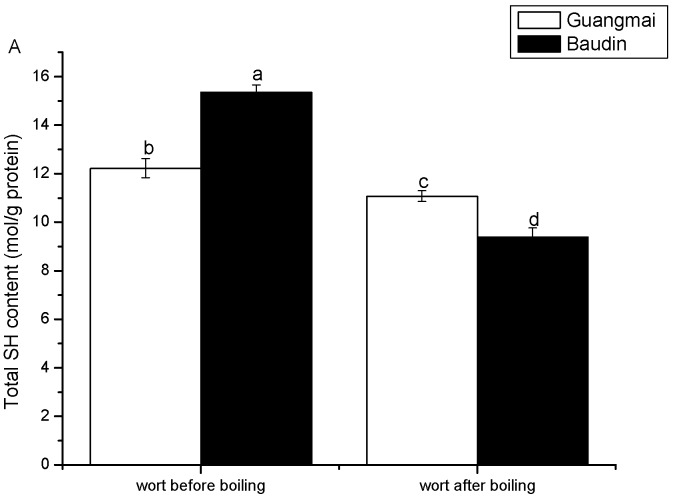
Total SH contents of wort proteins (before and after boiling) with the two varieties. Results are means and standard deviations of replicates. Different characters (a-d) on the top of a column indicate the significant differences (p<0.05) between wort proteins with the two varieties during the boiling.

**Figure 4:**
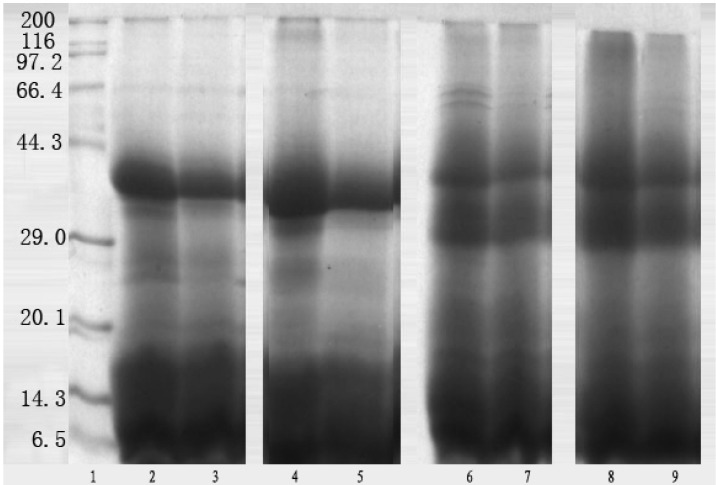
SDS-PAGE of wort proteins under reducing and non-reducing conditions: 1. Marker (kDa); 2. Baudin wort before boiling (with 2-ME); 3. Baudin wort after boiling (with 2-ME); 4. Guangmai wort before boiling (with 2-ME); 5. Guangmai wort after boiling (with 2-ME); 6. Baudin wort before boiling (without 2-ME); 7. Baudin wort after boiling (without 2-ME); 8. Guangmai wort before boiling (without 2-ME); 9. Guangmai wort after boiling (without2-ME).

Figure 3 shows that the total SH contents of wort proteins of the two varieties decreased after boiling. The results further confirmed the occurrence of boiling-induced protein unfolding [6] and subsequent aggregation/re-association of unfolded proteins. The boiling-induced changes in total SH contents were more pronounced in the Baudin worts, compared with the Guangmai ones, in accordance with the surface hydrophobicity results. As demonstrated by Perrocheau *et al*. [9], there was no reoxidation of cysteine in the 2D electrophoresis, because the protein sulfhydryls were alkylated immediately after boiling. It was thus reasonably concluded that the wort boiling process greatly affected the SH content and aggregation of those unfolded wort proteins would become prominent due to hydrophobic interactions, as illustrated by SDS-PAGE pattern (Figure 4).

### DSC thermal characteristics

The thermal properties of the wort proteins with the two varieties were evaluated by means of DSC, and the related DSC characteristics are summarized in Table 3. Wort proteins in the two varieties before boiling showed weak DSC endothermic response (data not shown) suggesting a large amount of components in wort proteins were unfolded and denatured. The DSC thermogram usually shows the protein denaturation as an endothermic peak, which is a net value of endothermic and exothermic processes occurring in a highly cooperative manner [25]. 

**Table 3:** DSC characteristics of wort samples with the two varieties^a^.

	T_o _(°C)	T_d_ (°C)	ΔH (J/g)	ΔT_1/2_ (°C)
**Baudin wort boiled before**	112.3	116.62	0.65	5.45
**Baudin wort boiled after**	111.44	115.53	0.47	6.79
**Guangmai wort boiled before**	111.39	115.91	1.06	5.98
**Guangmai wort boiled after**	111.84	115.09	0.98	6.61

^a ^Each value was the mean of duplicate measurements. The scan rate was 5°C per minute. The wort samples were dispersed at about 20% (w/v) in 50 mmol/L phosphate buffer (pH 7.0). T_o_, on-set temperature of denaturation; T_d_, thermal denaturation temperature; ΔH, enthalpy changes of endotherm and ΔT_1/2_, width at half peak height of endothermic peak. Values were expressed as the mean and standard deviations of triplicate measurements.

The results showed that the two wort proteins before boiling were of similar DSC patterns. The denaturation temperature (T_d_) can be used as a measure of thermal stability of proteins. However, there was a slight decrease in thermal denaturation temperature (T_d_) of the two kinds of wort proteins due to the presence of highly denatured protein in wort, implying that the proportion of undenatured protein or ordered structure found in wort between the two varieties were decreased upon boiling.

The boiling process caused a slight decrease in the enthalpy change (ΔH) of the endothermic peak of the two wort protein components (Table 3). The enthalpy value (ΔH) is correlated with the content of ordered secondary structure of a protein [26], as indicated by the proportion of undenatured protein. Thermal treatments could disrupt the chemical forces that maintain the structural integrity of protein molecules, such as hydrophobic and ionic interactions, hydrogen bonds and disulfide bonds, resulting in protein denaturation. Rupture of hydrogen bonds is considered an endothermic reaction which could increase the net endothermic contribution whereas the breakup of hydrophobic interactions and aggregation are exothermic reactions which could lower the net endothermic contribution resulting in a decrease in ΔH [27]. Thus, the boiling-induced unfolding of undenatured protein in worts and the exothermic reactions of aggregation as well as the breakup of hydrophobic interactions were more prominent since a partially unfolded protein would require less heat energy to denature completely than a native protein. As listed in Table 3, the ΔH value of Guangmai wort was higher than that of Baudin wort, which indicated that former had a much more ordered structure. On the other hand, as for the width at half-peak height of the major endothermic peak (ΔT_1/2_), which is an index of the cooperativity of the transition from native to denatured state [28], it slightly increased after boiling, thus revealing the loss of cooperativity in the denaturation process and the weakening of protein-protein interactions. Furthermore, similar DSC profiles were observed in wort protein before and after boiling for the two varieties, suggesting that little effect of variety difference on the changes of protein during boiling was obtained in this study.

### Electrophoretic separation

To elucidate the change of protein composition during wort boiling, SDS-PAGE was performed respectively under both reducing (adding mercaptoethanol to sample buffer) and non-reducing conditions (without mercaptoethanol). In the presence of reducing agent β-mercaptoethanol (2-ME), the patterns were similar between wort before boiling and after boiling of the two variety. An intense band of approximate 40 kDa was observed in the worts before and after boiling between the two varieties, correspondent to the protein Z, indicating that protein Z was a major component in wort. This result confirmed that protein Z could resist to heat denaturation and exist in beer [8,29]. Other bands were observed, including ~66 kDa, 25-29 kDa and 6.5-17 kDa in the Baudin wort before boiling, while a band of 116 kDa was also observed in Guangmai wort before boiling (Figure 4). In accordance with the study of Kordialik-Bogacka and Ambroziak [30], the 66 kDa band was detected in the two varieties in this study. However, the band intensities of 25-29 kDa and ~35 kDa in Baudin wort evidently decreased after boiling, while not only did the band intensities of 25-29 kDa and ~35 kDa decrease in Guangmai wort, but a band at 116 kDa also disappeared after boiling. The worts of both varieties displayed similar protein profiles after boiling under non-reducing conditions. The major protein band was also found at a molecular mass of 40 kDa. Moreover, the bands of 30-36 kDa and 6.5-20.1 kDa were detected in the non-reducing SDS-PAGE patterns of both worts before boiling. In addition, the Guangmai wort before boiling contained high molecular weight bands of 66.4-200 kDa, while the Baudin wort included ~66 kDa, ~60 kDa and 97.2-116 kDa ones, indicating the presence of disulphide bonds within their structures. After boiling, the band intensities of 66.4-200 kDa obviously decreased in the Guangmai wort under non-reducing conditions, while the band intensities of 97.2-116 kDa obviously decreased and the two bands of ~66 kDa and ~60 kDa disappeared in Baudin wort. The above results indicated that part of high molecular weight proteins dissociated into smaller ones due to reduction or denatured with the presence of hops and lead to their precipitation and removal, and that some low molecular weight protein formed high molecular weight protein via hydrophobic interactions or might be acylated and glycated through Maillard reactions with reducing sugar because the Maillard reaction still happens during the wort boiling to produce protein glycation [31] and further form precipitates with the hops. However, there was no evidence to support the contention that high molecular weight proteins were removed in noticeable quantities by heat denaturation during boiling, based on SDS-PAGE results. In this study it appeared that only small amounts were removed, in agreement with some recent reports [37,38,39], as similar protein patterns were observed in the two wort varieties before boiling and after boiling under reducing and non-reducing conditions, therefore little effect of variety difference on the protein changes during boiling was found in this study. 

**Figure 5:**
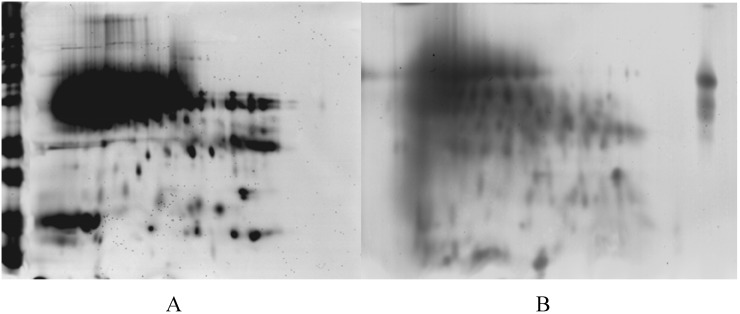
2-D gel protein patterns of wort protein before boiling (A) and after boiling (B). Proteins were separated in the first dimension on a IPG strip pI 3-10 and in the second dimension on a 15% acrylamide SDS gel. The proteins were silver stained. (Protein Markers: 116, 90, 75, 50, 37, 25, 20, 15 and 10 kDa)

In order to characterize exactly the composition of wort proteins, these were analyzed using 2-D analysis. However, the 2-D patterns of Guangmai wort proteins were poorly resolved due to the high sugar content, thereby only Baudin wort proteins were investigated in this study. The observed changes in the protein patterns displayed in the silver-stained gels could be related to the events occurring in boiling. Figure 5 shows the 2-D gels of Baudin wort protein before and after boiling. As expected, clear differences could not be extracted from the 2-D maps. Approximately 120 well-defined spots could be detected on each wort gel in the pI range of 3-10 using the 2-D gel analysis software Melanie. It was noteworthy that the protein profiles of the wort proteins before and after boiling were quite similar. Moreover, the wort before boiling gel displayed a much higher number of spots in the pI range of 5-8, mass of 12-40 kDa (Figure 5A), in accordance with the study of Gorinstein [19]. It was observed that a significant decrease in the number of protein spots in the range of 10-20 and 35-40 kDa and in the pI range of 5-8 for wort protein after boiling, in accordant with SDS-PAGE results. However, a slight increase in the number of protein spots in the range of 75-90 kDa and in the pI range of 4-6 for wort protein after boiling was detected in 2-D patterns, indicating that the formation of a small amount of soluble aggregates via hydrophobic interactions. As shown in Figure 5, a large, intense staining spot was observed at around an isoelectric point (pI) of 4-6 and a molecular mass of 35-60 kDa in all of the 2-D gels. Presumably this gel region included protein Z, which has coincident characteristics with this region. Based on the study of Perrocheau [9], it was speculated that protein Z (40 kDa), LTP1 (10 kDa) and α-amylase inhibitor (12-16 kDa) could resist heat denaturation during boiling and exist in beer. However, further research is required to elucidate the relationship of wort protein composition and beer quality using immunological method. The improved understanding of the impact of boiling process on each malt protein will potentially provide further scope for optimizing both malt characteristics and beer quality.

### Gel filtration chromatography analysis of wort proteins

**Figure 6:**
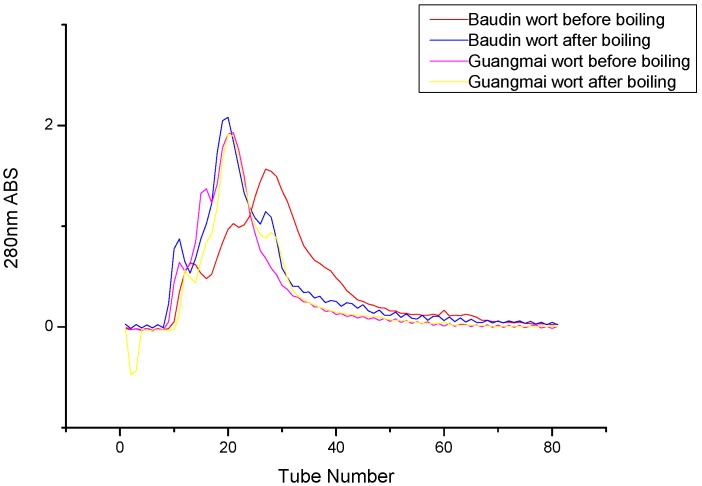
Gel-filtration protein profile of Baudin and Guangmai wort before and after boiling.

**Figure 7:**
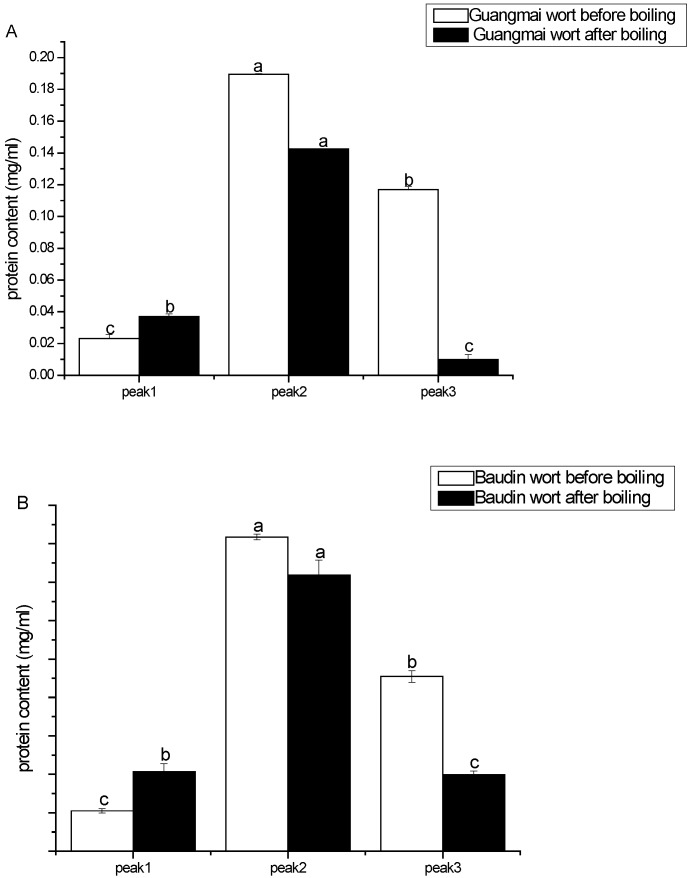
Protein contents of the peaks I, II and III from Guangmai (A) and Baudin (B) worts before and after boiling.

The SEC elution profiles of the wort proteins with the two varieties during the boiling were investigated, using 50 mmol/L acetate buffer (pH 5.8) containing 0.02% NaN_3_ as the eluting solvent. During the boiling, Sephacryl S-200 High Resolution gel filtration chromatography fractioned the wort protein into incompletely resolved peaks. As shown in Figure 6, the proteins showed similar profile patterns. For each malt variety, the chromatograms were divided into three parts and there were three components in wort proteins during the boiling. However, the obtained SE-HPLC curves were different from those reported by Osman *et al*. [20] with eight peaks of varying magnitudes and degree of separation due to different malt variety and gel filtration chromatography column used. After boiling, the area of the intermediate-size peak (peak II) increased and the peak position moved backwards concomitantly with a decrease in the low molecular mass peak (peak III), while the shape and position of peak I remained unchanged. This was attributed to the fact that the smaller proteins formed medium and high molecular weight proteins via hydrophobic interactions or oxidation and further form precipitates with hops at the same time the components of the intermediate-size peak (peak II) dissociate into small-size ones. The quantitative analysis of the proteins recovered in the fractions, using the Bradford Coomassie blue dye binding assay (Figure 7), revealed that the protein contents in peak II and III decreased while that of peak I slightly increased after boiling, revealing the formation of a small amount of soluble aggregates.

Furthermore, it is pertinent to add that the peak II fraction was coloured (yellow), while the other peak fractions showed limpidity or were slightly yellow, due to the combination of phenolic and polyphenolic groups with sugars and proteins through Maillard reactions [20]. 

Some of the materials from the peaks of SE-HPLC were collected and electrophoresed in the SDS-PAGE system (data not shown here), but Commassie Blue reagent staining of the gels failed to detect any permanent bands after the destaining of peak III. No attempts were made to use other reagents at this stage. Peak II in the two kinds of worts represented the protein with the molecular mass of 40 kDa and the corresponding band intensity reduced after boiling. A fainter band of ~ 66 kDa was detected in peak I and disappeared after boiling. Moreover, the same electrophoretogram appeared in the two kinds of worts. This evidence confirmed the above SDS-PAGE analysis that 40 kDa protein could resist heat denaturation and exist in the final product (beer) and that part of the high and middle molecular weight protein might form small amounts of soluble aggregates after boiling. Futhermore, little effect of variety difference on the changes of protein during boiling was also found due to very similar gel filtration chromatogram profiles. 

### CD analysis

**Figure 8:**
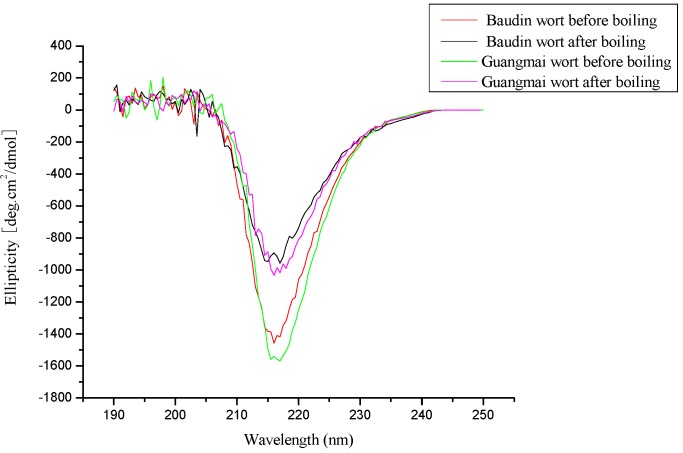
The CD spectra for wort protein before and after boiling between the two varieties

The influence of boiling on the secondary structure of wort protein between the two varieties were evaluated by far-UV CD analysis. The CD spectra for wort protein before and after boiling were very similar between the two varieties (Figure 8), indicating that the effect of variety difference on the changes of protein during boiling was insignificant. 

The CD spectra for wort protein before between the two varieties showed similar strong negative band around 216 nm, which was characteristic of β-sheet structure. However, boiling significantly reduced the band intensity at 216 nm, which was indicative of a random structure and confirmed that boiling effectively disrupted the α-helix secondary structure.

The α-helix, β-sheet, and random coil content of wort protein before and after boiling were estimated for the two varieties (Table 4). The results showed that α-helix contents were reduced, β-sheet and β-turn contents were slightly increased, and random coil contents were markedly increased for both varieties after boiling, indicating a partial loss of the α-helix structure and thereby partial unfolding of the wort protein and that some retained their secondary structures throughout the brewing process. Moreover, the data confirmed the results of DSC indicating that unfolding started on mashing and was achieved on boiling.

**Table 4:** Comparison of secondary structure contents of wort protein before and after boiling between the two varieties ^a^.

	Baudin wort		Guangmai wort	
	before boiling	after boiling	before boiling	after boiling
**α- helix (** **％)**	30.1	22.8	29.8	22.1
**β- sheet (** **％)**	14.7	15.2	14.9	15.5
**β- turn (** **％)**	14.4	14.9	14.5	14.9
**Random coil (%)**	40.8	47.1	40.8	47.5

^a ^Each value was the mean of duplicate measurements.

## Conclusions

In conclusion, the study has confirmed that the boiling greatly influences the physicochemical properties and structure of wort proteins. Boiling results in a decrease in protein and amino acid contents. Boiling also results in the decrease in surface hydrophobicity and free sulfhydryl content and enthalpy value, indicating protein unfolding and denaturation, as well as the formation of a small amount of soluble aggregates via hydrophobic interactions during the boiling. Two-dimensional electrophoresis and SE-HPLC analysis reveal that major protein component 40kDa in wort proteins is able to resist heat denaturation, while confirming wort protein denaturation and the formation of a small amount of soluble aggregates via hydrophobic interactions. Results from CD analysis show that boiling disrupts the secondary structure to partial unfolding. Additionally, this study has shown that the effect of wort boiling on protein properties is independent of malt variety. Further studies should be carried out to reveal the conformational changes in protein structure during the boiling and to correlate the changes of protein during the wort boiling with beer quality.

## Experimental

### Worts and preparation of wort protein

Worts were produced from Baudin and Guangmai malts on an industrial scale. 100% of the malt used in each case was from a single barley cultivar. Similar mashing and boiling procedures were used. The wort samples of Baudin and Guangmai before and after boiling were provided by Zhujiang Brewery Group Co., Ltd., P.R. China. Wort proteins before and after boiling were extracted by adding solid ammonium sulfate to the wort to achieve a relative saturation of 80%. The precipitate formed overnight at 4 °C was collected by centrifugation (7000×*g*) for 10 min, then the supernatant was discarded and the pellet was resuspended in water. Afterwards, the mixture was homogenized to ensure the pellet to be completely broken up. The samples were dialyzed and lyophilized before storage at 4 °C until use. The protein contents of the products were determined by means of the micro-Kjeldahl method (N×6.25). Measurements were performed in duplicate.

### Analysis of amino acids

The lyophilized protein was hydrolyzed in a sealed tube for 24 h with 6 mol/L HCl at 110°C, then the amino acid composition was analyzed by high-performance ion-exchange chromatography and post-column derivatization using *o*-phthalaldehyde and sodium hypochlorite using a Waters amino acid analyzer equipped with a PICO・TAG column. The determination was carried out at 38°C, with a detection wavelength of 254 nm, and a flow rate of 1.0 mL/min. Tryptophan was not determined.

### Measurement of surface hydrophobicity (H_o_)

H_o_ was determined using ANS, according to protocol of Kato and Nakai [32]. Fluorescence intensity (FI) was measured at wavelengths of 390 nm (excitation) and 470 nm (emission) using a F-4500 spectrofluorometer at ambient temperature, with a constant excitation and emission slit of 5 nm. The initial slope of the FI versus protein concentration plot was calculated by means of linear regression analysis and used as an index of H_o_. Measurements were performed in triplicate.

### Measurement of free sulfhydryl (SH) content

The amount of free sulfhydryl of lyophilized wort protein was measured with a colorimetric method, as modified by Chan and Wasserman [33]. Samples (≈15 mg) were suspended in reaction buffer A, consisting of 8 mol/L urea, 3 mmol/L EDTA, 1% SDS, and 0.2 mol/L Tris-HCl (pH 8.0, 1.5 mL). Samples were vortexed for 30 sec and placed on a constant agitation shaker at room temperature. After 1 hr, buffer B (0.15 mL, 4 mg DTNB dissolved in 1 mL of 0.2 mol/L Tris-HCl) was added to each sample, and shaking continued for another 1 hr. Samples were then centrifuged at 13,600×*g* for 10 min at room temperature, and the absorbance of the supernatant was read at 412 nm against a blank consisting of 1.5 mL of buffer A and buffer B. Each sample was determined in triplicate. 

### Differential Scanning Calorimetry

The thermal denaturation of wort proteins were examined using a TA Q100-DSC thermal analyzer (TA Instruments, New Castle, DE), according to the procedure of Meng and Ma [34], with some modifications. Approximately 2.0-3.0 mg protein samples were accurately weight into aluminum liquid pans, and 10 μL 50 mmol/L phosphate buffer (pH 7.0) was added. The pans were hermetically sealed and heated from 20 to 160 °C at a rate of 5°C/min. A sealed empty pan was used as a reference. Peak or denaturation temperature (T_d_) of different protein components and denaturation enthalpy change (ΔH) and width at half peak height of endothermic peaks (ΔT_1/2_) were computed from the thermograms by the Universal Analysis 2000, version 4.1D software (TA Instruments-Waters LLC, USA). All experiments were conducted in triplicate. In all cases, the sealed pans containing wort protein samples and buffers were equilibrated at 25°C for more than 6 h. 

### Gel filtration chromatography

Gel filtration chromatography was performed by using Waters 650E Advanced Protein Purification System and a Sephacryl S-200 High Resolution column (GE Healthcare UK Limited, Φ 2.6 cm×60 cm). Wort protein aliquots containing 300-350 mg of protein were applied to the column. Elution rate was 0.8 mL/min using 50 mmol/L acetate buffer (pH 5.8) containing 0.02% NaN_3_ in deionized water. Fractions of 6 mL were collected and the absorbance at 280 nm was measured. The column was washed after each sample with 0.1 mol/L NaOH, distilled water and re-equilibrated before loading the next sample.

### Electrophoretic analyses

Sodium dodecylsulfate-polyacrylamide gel electrophoresis (SDS-PAGE) was done as described by Laemmli [35] on a discontinuous buffer system with resolving gels (total acrylamide [T] 15%, cross-linking [C] 2.7%) and a stacking gel (T 4%, C 2.7%) by using a Mini-Protean II electrophoresis unit (Bio-Rad). Samples were dissolved in reducing sample buffer (5 mol/L urea, 4% SDS, Tris buffer pH 8.0, containing 1% 2-mercaptoethanol) and non-reducing sample buffer (without 2-mercaptoethanol). Separations were carried out at 15 mA in the stacking gel, and at 25 mA in the resolving gel. The gel was stained with Coomassie Brilliant Blue R-250 in 50% trichloroacetic acid and destained in 7% acetic acid [methanol/acetic/water, 227:37:236 (v/v/v)]. For 2-D electrophoresis the freeze-dried protein samples were completely dissolved in 8 M urea, 2% w/v CHAPS, 2% v/v IPG buffer pH 3-10 (Amersham Biosciences, Uppsala, Sweden), 0.01% bromophenol blue, 20 mM DTT, and were centrifuged at 12,000 rpm for 10 min. IEF was performed using a Pharmacia Biotech IPG phor electrophoresis system (Amersham Biosciences). Prior to second dimension development, the IPG strips were equilibrated for 10 min in equilibration buffer (50 mM Tris/HCl pH 8.8, 6M urea, 30% v/v glycerol, 2% w/v SDS, 0.01% bromophenol blue) containing 10 mg/mL DTT, followed by 10 min in equilibration buffer containing 25 mg/mL iodoacetamide. The second dimension was run on 15% acrylamide gels on a vertical system. Gels were stained with silver nitrate [21, 22].

### Circular Dichroism Spectroscopy

The secondary structure of proteins was determined by CD at 25 °C in the far UV (from 190 to 250 nm) using a CD6 Jobin-Yvon dichrograph. Proteins were solubilized in a final concentration of 0.5 mg/mL. A quartz cell of 0.2 nm path length was used. Data were expressed as mean-residue ellipticity. Secondary structures were determined using the CONTIN software.

### Statistical analysis

An analysis of variance was performed on the data, and a least significant difference test with a confidence interval of 95% was used to compare the means.
